# Essential elements to “design for dissemination” within a research network—a modified Delphi study of the Community-Academic Aging Research Network (CAARN)

**DOI:** 10.1186/s43058-021-00122-z

**Published:** 2021-02-12

**Authors:** Maria C. Mora Pinzon, Shannon Myers, Jill Renken, Erin Eggert, Betty Chewning, Jane E. Mahoney

**Affiliations:** 1 Department of Medicine, Division of Geriatrics and Gerontology, School of Medicine and Public Health, University of Wisconsin – Madison, Madison, WI USA; 2 Department of Family Medicine and Community Health. School of Medicine and Public Health, University of Wisconsin - Madison, Madison, WI USA; 3 Wisconsin Institute for Healthy Aging, Madison, WI USA; 4 School of Pharmacy, University of Wisconsin – Madison, Madison, WI USA

**Keywords:** Implementation science, Purveyor, Community participation, Community-based participatory research

## Abstract

**Background:**

The Community-Academic Aging Research Network (CAARN) was developed in 2010 to build partnerships, facilitate research, and ultimately accelerate the pace of development, testing, and dissemination of evidence-based programs related to healthy aging. CAARN has facilitated development and testing of 32 interventions, two of which are being packaged for scale-up, and three of which are being scaled up nationally by non-profit organizations. The purpose of this study is to describe CAARN’s essential elements required to replicate its success in designing for dissemination.

**Methods:**

We conducted a modified Delphi technique with 31 participants who represented CAARN’s organization (staff and Executive Committee) and academic and community partners. Participants received three rounds of a web-based survey to rate and provide feedback about the importance of a list of potential key elements compiled by the authors. The criterion for establishing consensus was 80% of responses to consider the element to be extremely or very important.

**Results:**

Response rate was 90% in Round 1, 82% in Round 2, and 87% in Round 3. A total of 115 items were included across rounds. Overall, consensus was achieved in 77 (67%) elements: 8 of 11 elements about academic partners, 8 of 11 about community partners, 29 of 49 about the role of the community research associate, 16 of 21 about the role of the director, 9 of 17 about the purveyor (i.e., the organization that scales up an intervention with fidelity), and 7 of 7 about the overall characteristics of the network.

**Conclusions:**

The development of evidence-based programs designed for dissemination requires the involvement of community partners, the presence of a liaison that facilitates communications among academic and community stakeholders and a purveyor, and the presence of a pathway to dissemination through a relationship with a purveyor. This study delineates essential elements that meet the priorities of adopters, implementers, and end-users and provide the necessary support to community and academic partners to develop and test interventions with those priorities in mind. Replication of these key elements of the CAARN model may facilitate quicker development, testing, and subsequent dissemination of evidence-based programs that are feasible to implement by community organizations.

**Supplementary Information:**

The online version contains supplementary material available at 10.1186/s43058-021-00122-z.

Contributions to the literature

- Describes the necessary support that community-academic partnerships need to develop and test interventions that are designed for dissemination.
- Delineates the importance of a relationship with a purveyor. A purveyor is defined as an organization that actively works to distribute an evidence-based program or practice in such a way that it can be implemented with fidelity and good effect at an implementation site.
- Describes the activities that a liaison does to support effective communications between partners from diverse backgrounds.

## Introduction

The Community-Academic Aging Research Network (CAARN) is a research network that was created in 2010 within the University of Wisconsin – Madison to serve as a bridge between researchers and community organizations in Wisconsin serving older adults. CAARN’s goal is to facilitate research partnerships to develop new health behavior change interventions and take them through the stages of research, culminating in evidence-based programs packaged in a feasible way for adopter organizations to implement and for a dissemination partner or purveyor to take to scale [1].

CAARN has facilitated 32 projects, corresponding to 47 academic investigators and 50 Wisconsin counties and 1 tribe, garnering 55 grants totaling $20 million in extramural and $3 million in intramural funding. These projects have been developed within the state of Wisconsin; when necessary, research has expanded to include partners in neighboring states (e.g., Minnesota). CAARN’s processes have resulted in three proven self-efficacy-based health behavior change interventions that are now being disseminated nationally [2–4], and two are being packaged for dissemination [5–7]. Currently, four interventions are being tested in a randomized clinical trial after a successful CAARN pilot, four interventions are applying for funding for efficacy studies, four have completed pilot or feasibility studies, and five are applying for funding for their pilot studies.

To our knowledge, while community-academic partnerships and incubators exist at many academic institutions in the United States of America (US), CAARN is the only infrastructure that includes dissemination of products as a core function. CAARN applies the principles of “design for dissemination” across its network. Design for dissemination is defined as the process of ensuring that evidence-based interventions are developed in ways that match adopters’ needs, assets, and time frames [8]. Understanding the needs and capacities of adopters can be achieved through involving them in the design and testing of interventions and through partnering with a purveyor who regularly works with similar adopting organizations. CAARN works with the Wisconsin Institute for Healthy Aging (WIHA), a not-for-profit organization dedicated to disseminating evidence-based healthy aging programs and supporting their implementation in communities throughout Wisconsin and across the nation. As a purveyor, WIHA scales up evidence-based programs across the state and the nation by assisting community organizations to adopt the programs, certifying facilitators who deliver the evidence-based programs under the auspices of the community organizations, and monitoring fidelity of the programs once they are implemented.

The purpose of this study was to define and understand the key elements that have been responsible for the network’s success in achieving its goals: developing health behavior change interventions that are effective and feasible to scale up, and ensuring hand-off of packaged interventions to a purveyor for broad scale-up. We used the modified Delphi method to identify essential elements, which we defined as those that were extremely important or very important to achieve the goals of a research network like CAARN. This information is important because it helps us to understand the components that CAARN should maintain as it expanded to bring on new community partners, and to provide generalizable information that may help others to enhance the capacity of their community-academic research networks to facilitate the broad scale-up of research products into practice.

## Methods

### Overview of CAARN

From its inception in 2010 until mid-2019 when it expanded through funding from the National Institute of Health, CAARN’s infrastructure consisted of a Faculty Director, a Program Manager, and two Community Research Associates (CRAs). The roles and responsibilities of each partner and the processes involved in the development of projects are described in a previous publication by Mahoney et al [1]. In brief, the CRAs serve as liaisons, facilitating communication and collaboration between the academic investigator and the community partner organization, assuring that community input is included in all stages of the study. At the time of survey, the CRA was a community representative, who had been hired by the purveyor, working 50% of the time for CAARN, and the remaining 50% for the purveyor, providing services to facilitate community organizations’ adoption, implementation, and sustainment of evidence-based programs [1].

An Executive Committee (EC) composed of community and academic stakeholders oversees CAARN’s month-to-month activities and prioritizes potential research projects based on community need, current capacity of community stakeholders and CRAs, and the likelihood of gaining research funding. Until 2019, community organizations that participated in CAARN belonged to Wisconsin’s Aging Network, which is comprised of a diverse mix of organizations that are responsible for delivering Older Americans Act and Wisconsin Elders Act programs and services to Wisconsin’s older adults. Since the 2019 survey, four additional network partners, each employing a 50% CRA, have been added: three community organizations serving African-American older adults, and a quality improvement organization serving Wisconsin’s healthcare systems. These additional network partners have been added to diversify the organizations that can partner with CAARN and better address health disparities that burden African-American/Black communities in WI and the US.

As described before, all of CAARN projects are designed with the ultimate aim of broad dissemination. Once interventions are packaged for dissemination, a purveyor organization distributes them for adoption and implementation by organizations across the US. WIHA has been the purveyor for four of the five interventions that have reached the dissemination stage [2, 4–7]; the other intervention is being disseminated by another organization that has greater expertise in delivering similar interventions and better infrastructure to support the program’s distribution.

### Overview of modified Delphi method

The Delphi method is commonly used to identify essential elements in complex issues by relying on experts anonymously completing a series of questions over subsequent rounds until consensus is achieved [9, 10]. In the original Delphi, the process starts with a series of open-ended questions [11]. A modified Delphi technique starts with a set of items selected by the organizers, which are then rated for importance over a series of anonymous surveys by experts who each additionally provides the rationale underlying their choice of rating [11]. The Delphi method relies on providing participants with a summary of the ratings and comments on ratings from the previous rounds and does not require in-person meetings to achieve consensus [11–13].

### Selection of Delphi panelists

The Delphi survey targeted three types of experts: (1) CAARN organization (current and former CRAs and EC members); (2) community partners (members of community organizations that have participated in at least one research project facilitated by CAARN in the last 5 years); and (3) academic partners (research faculty who have participated in at least one research project facilitated by CAARN in the last 5 years). An email inviting participation in the Delphi study was sent by author MP to all current and former CRAs (3 individuals), all current EC members (4 individuals), and current and former partners that matched the above criteria (42 academic partners, 71 community partners). The decision to invite only current EC members was because they were familiar with the current functioning of CAARN when the Delphi was completed; EC members were representatives from state and regional organizations serving older adults; they were not paid to serve on CAARN’s executive committee.

The email described the goal and overall process of the Delphi study and invited participants to complete an online form confirming their agreement to participate. No monetary incentive was described in the invitation email. This study was exempted by the UW-Madison Institutional Review Board.

Twelve academics (12/42, 29%), 21 community partners (21/71, 29%), and all seven of CAARN’s staff/EC that were invited (3/3 CRAs, 4/4 EC members, 100%) agreed to participate. To assure an equal representation of partners, all academic partners who agreed were included. A sample of 12 community partners was selected by authors SM and MP, with preference given to those that had longer experience working with CAARN (i.e., had partnered with CAARN in more than one research project, had participated in a long-term project) or had participated in at least one project as an adopter (person in the organization making the decision to partner on the research project) and an implementer (person within the organization directly taking part in the research project, either through recruiting participants, delivering the intervention). After ranking community partners based on their extent of engagement, those 12 with more exposure to CAARN were invited to participate. We included a mix of both adopters and implementers to represent the variety of perspectives of community partners. The final panel of experts consisted of 12 academic partners, 12 community partners, and 7 CAARN organization members staff (3 CRAs and 4 EC members). If panelists did not complete the survey they were not invited to participate in subsequent rounds, as described in previous protocols [14]. To incentivize participation in Round 3, participants from community organizations were offered a monetary incentive in the invitation for Round 3 ($50).

### Selection of Delphi questions for Round 1

The research team, which consisted of CAARN’s research scientist (MP), an academic partner who had worked with CAARN on 3 research projects (BC), two current and one former CRAs (JR, EE, SM), and CAARN’s director (JM), developed the candidate items for the Delphi survey. The CRAs who were invited to complete the surveys participated in the development of the Delphi to assure the questions avoided jargon, were formatted to be meaningful to community partners, and encompassed different aspects of the research process. The questions for Round 1 of the Delphi survey were derived from (1) informal interviews with four stakeholders; (2) discussions among the research team; and (3) input from CAARN’s EC. Stakeholder interviews, performed by author MP, consisted of four unstructured interviews with two current academic and two community partners working with CAARN to gather their impressions of CAARN as a research network and their perception of the elements that were essential to achieve its goals. Stakeholders that participated in interviews also received invitations to participate in the Delphi surveys, but to maintain anonymity, we do not know if they accepted those invitations and participated in the Delphi process.

To minimize bias, no questions were eliminated in the development process unless they were considered a duplicate. The resulting elements were grouped into six categories. These included function of the *Overall Network*, and characteristics and function of each of its following components: *Community Research Associate*, *Network Director*, *Purveyor*, *Academic Partner*, and *Community Partner*. The authors and the EC reviewed a draft of the survey to assure clarity of the questions and adequate description of the intent of the survey.

### Delphi process

The modified Delphi technique was conducted over three rounds from November 2018 to June 2019. All rounds of the survey were fielded online, using Qualtrics software (Qualtrics, Provo, UT, USA. https://www.qualtrics.com). Panelists were sent an email containing an individualized link to each survey, which was available for 4 weeks. Reminder emails were sent to non-responders on a weekly basis until a total of 5 reminders per round were sent.

With each survey, panelists were provided the following definition of CAARN’s stated goal: “To facilitate research partnerships that can take health education interventions through the stages of research, culminating in an evidence-based programs packaged for dissemination that are effective and feasible to implement by organizations.” Panelists were provided a list of potential key elements and were asked to rate the importance of each of the items to the effectiveness of CAARN in achieving its stated goal, using a 5-point Likert response scale: not at all important, slightly important, moderately important, very important, and extremely important.

Consensus was defined as achieving an 80% agreement in the responses, similar to other studies [15, 16]. This threshold was applied in the following way: we combined the two low-importance responses (*not at all/slightly important*) into one group and the two high-importance responses (*extremely/very important*) into another group. If at least 80% of panelists selected either *not at all/slightly important* or *extremely/very important*, the item was considered as having consensus and was not included in subsequent rounds. Further, if the consensus fell on the high-importance end of the scale (*extremely/very important*) the item was deemed to be a key element (i.e., essential to CAARN’s effectiveness).

Round 1 consisted of 106 Likert-type items, and 12 open-ended items for panelists to provide comments and suggest new items for Round 2. Opportunities to suggest new items were offered only in Round 1. All comments for items for which there was no consensus and all suggestions for new items were included in Round 2, unless the item was considered a duplicate. After Round 1, items that achieved consensus were removed and the authors reviewed the remaining items to assess if clarification or new wording was necessary for any items that did not achieve consensus (i.e., where definitions for elements could be considered ambiguous).

Round 2 included all Likert-type items that did not achieve consensus in Round 1 and the new items suggested by the panelists. For each item, the following elements were included: statement as presented in Round 1, panelists’ aggregated responses from Round 1, a re-statement of the question for Round 2, the Likert scale, and an open-ended field for panelists to explain their reasoning for their selection. If the question was modified to improve clarity between Round 1 and Round 2, the statement “this question was rewritten for clarification purposes” was included before the question. (For example, the definition of a “stakeholder” was added in Round 2 to clarify the roles that stakeholders can take in research.) After Round 2, items that achieved consensus were removed and the authors reviewed the remaining items to assess if clarification or new wording was necessary for any items that did not achieve consensus.

Round 3 included all items that did not achieve consensus in Round 2 but were considered *extremely/very important* by at least 50% of panelists. Items where fewer than 50% of the panelists agreed that the element was extremely or very important were removed from Round 3 because it was deemed unlikely that the element would meet the consensus threshold of 80% through further rounds. Each Round 3 item included a statement as presented in Round 2, panelists’ aggregated responses from Round 2, all the comments received in Round 2, a re-statement of the question for Round 3, the Likert scale, and a new field for comments. If the question was modified between Round 2 and Round 3, the statement “this question was rewritten for clarification purposes” was included before the question and the change was underlined for easier understanding.

For any item failing to achieve consensus on the high- and low-importance ends of the scale after Round 3, a Spearman’s rank correlation coefficient was calculated to determine the strength of the correlation between the second and third rounds’ responses. A moderate or strong positive Spearman’s correlation (Coefficient > 0.29) between the responses to an item comparing Rounds 2 and 3 indicated that responses did not change significantly between rounds, therefore triggering the termination of the item from the survey because of the low likelihood that it would achieve consensus in a fourth round. For elements that had weak Spearman’s rank (Coefficient 0.1–0.29), authors MP and JM reviewed the comments received in Rounds 2 and 3 to assess the need for subsequent rounds [15]. An item was terminated if there were no new comments in Round 3 compared to Round 2, as this meant there would be no additional information to affect panelists’ decision-making in subsequent rounds.

### Data analysis

Descriptive statistics included frequency tables. Authors performed a secondary analysis of the responses to describe variations according to the characteristics of the panelists. For this process, each panelist’s responses were converted to a numerical scale (5 = extremely important, 4 = very important, 3 = moderately important, 2 = slightly important, 1 = not at all important). The Shapiro test was used to check the assumption of normality. All samples were determined to be non-normal. Therefore, mean and median scores per type of panelist were calculated for each item and the Shapiro test was used to check the assumption of normality for each variable. All samples were determined to be non-normal and a Wilcoxon Mann-Whitney test was used to identify differences among the types of panelists. All *p* values were 2-tailed and *p* < 0.05 was used to determine statistical significance. SAS 9.3 was used to conduct statistical analyses (SAS Institute, Inc. Cary, North Carolina).

## Results

### Round 1

**Fig. 1:**
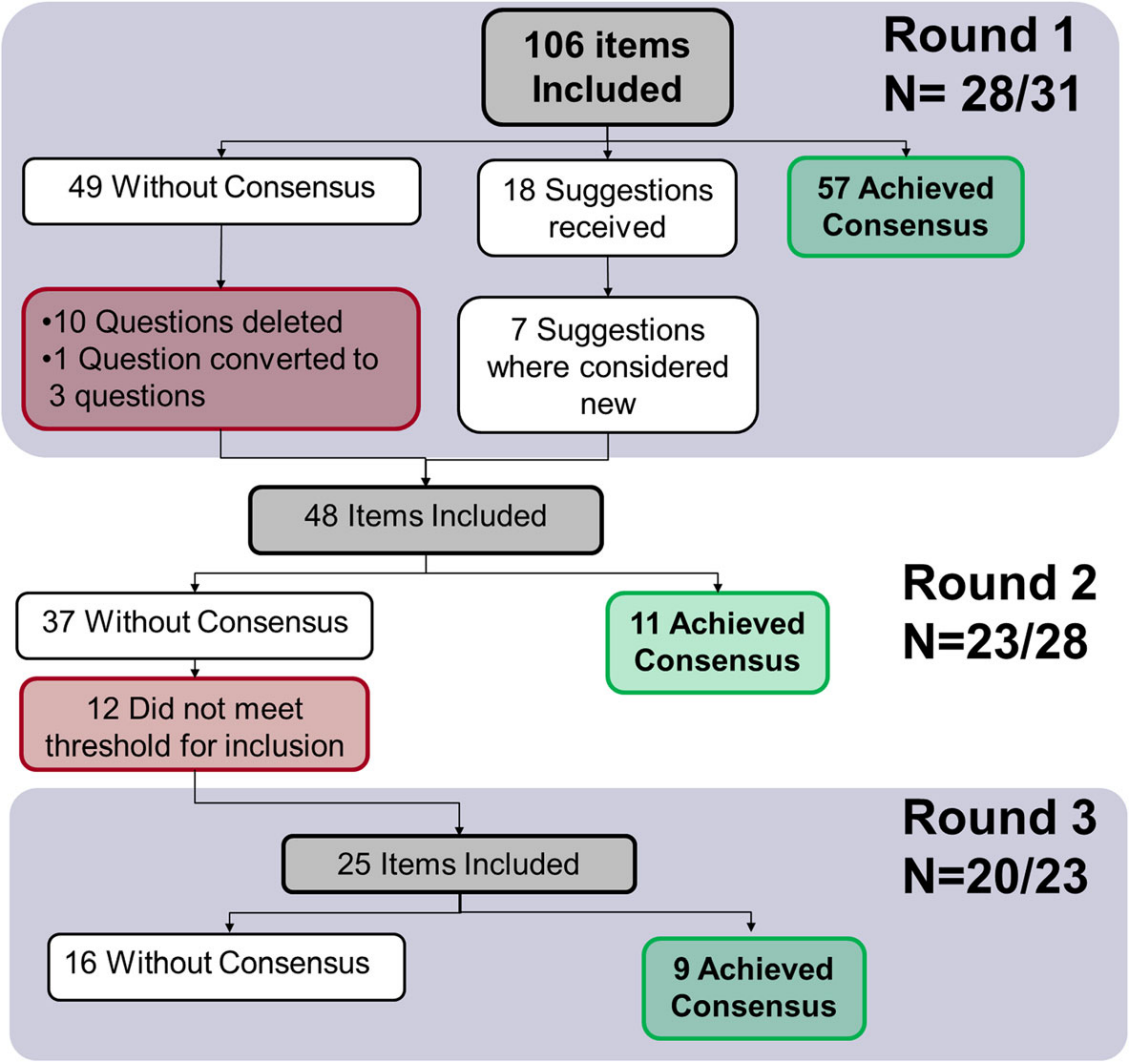
Overview of the Delphi rounds

Of the 31 individuals who agreed to participate in Round 1, 28 (90%) completed the survey. Regarding their experience working with CAARN, 48% of panelists had worked in 2–3 projects, 28% in one project, and 17% in 4 projects or more. Table 1 shows the distribution according to type of panelist. In Round 1, 57 items out of 106 achieved consensus and were considered essential (Table 2), and 49 did not achieve consensus. At the review of results, the research team determined that 10 questions were considered ambiguous or duplicative and were deleted, 26 questions were reworded to improve clarity, and one question was split into three questions. Additionally, 18 comments were received, but only seven suggested new items for inclusion in Round 2 (Fig. 1). A total of 48 items were therefore submitted to Round 2.

**Table 1:** Response rates by survey round and expert type

Expert	Invited	Round 1	Round 2	Round -3
		*n* (column %)	*n* (column %)	*n* (column %)
Community partners	12 (39%)	12 (43%)	9^a^(39%)	7 (35%)
Academic partners	12 (39%)	9 (32%)	9 (39%)	9 (45%)
CAARN staff and EC members	7 (22%) (3 CRA, 4 EC members)	7 (25%)	5 (22%)	4 (20%)
**Totals responses (*n*, % of panelist that responded**^**b**^**)**	**31 (100%)**	**28 (90%)**	**23 (82%)**	**20 (87%)**

^a^2 panelists left the invited organizations and contact information was not available

^b^ Calculated dividing the number of panelists that completed the survey by the number of panelists that received an invitation for that round (Round 1: 28/31, Round 2: 23/28, Round 3: 20/23)

**Table 2:** Items that are considered essential to perform the work of a research network like CAARN

Essential elements *(Arranged in descending order of percentage in final agreement)*	Round when consensus was achieved	Percent of panelists that considered the element as essential		
		Round 1	Round 2	Round 3
**Overall characteristic of the research network**				
• Aligns research projects with the needs of community partners	1	96%		
• Has access to a network of community/local organizations	1	96%		
• Usefulness and feasibility of health interventions is tested in the early stages of research	1	96%		
• Has a relationship with an organization(s) that want to distribute the research products (i.e., the proven interventions) after research is completed^ab^	3	69%	66%	95%
• Has a Program Manager/Administrator	1	93%		
• Have a community research associate (CRA) on staff^a^	2	78%	91%	
• Has a goal to move projects along the research continuum (e.g., Pilot ➔ Small clinical trial ➔ Randomizes clinical trial ➔ Dissemination)	1	89%		
• Have a Community-Academic Executive Committee, which is formed by community and academic stakeholders. Its functions include to prioritize studies, oversee identifying and selecting community and academic partners for research teams, and problem solving for research partnerships^a^	2	67%	83%	
• Having a group of individuals who are potentially interested in using the resulting health interventions in the future.	1	82%		
**Regarding the academic partners invited to participate in CAARN projects (Principal Investigator or their mentor on the grant if they are junior)**				
• Has good communication skills	1	96%		
• Is open to receive feedback	1	93%		
• Is open to learn new concepts (e.g., D&I concepts, community-based participatory research)	1	93%		
• Is committed to move projects along the research continuum (e.g., Pilot ➔ Small clinical trial ➔ Randomizes clinical trial ➔ Dissemination)	1	93%		
• Principal Investigator (or their Mentor on the grant, if they are junior) has been successful in obtaining grant funding^a^	2	74%	91%	
• Has good writing skills	1	89%		
• Considers community partners as equals in the research team	1	89%		
• Has knowledge of the principles of dissemination and implementation research (e.g., design for dissemination, reach, fidelity, context, among others)^a^	3	79%	76%	80%
**Regarding the community partners invited to participate in CAARN projects. Community Partners are defined as those in the community working with the researchers/CAARN to implement the intervention/program**				
• Have staff who are enthusiastic	1	100%		
• Have capacity (time, staff, resources) to participate in research	1	96%		
• Has a champion for the project within the organization	2	n/a	95%	
• Have strong, collaborative, and productive working relationships within community	1	89%		
• Participation in research activities has support from leadership	1	86%		
• Receive financial support from the research project to compensate for time/staff that is dedicated to research related activities	1	86%		
• Has regular communications (e.g., meetings, symposiums, conference calls) with similar agencies to share experiences and current initiatives^a^	3	57%	62%	85%
• Be invited to provide input in all phases of the study (e.g., intervention design, study design, grant writing, data collection, interpretation of results) ^ab^	3	78%	76%	80%
**Regarding the Community Research Associate (CRA)** *(According to the National Institutes of Health, a CRA is a community representative that serves as a primary liaison facilitating communication and collaboration between the academic health center and the local community)*				
• Has good communication skills	1	100%		
• Has a positive attitude	1	100%		
• Has excellent organizational skills	1	100%		
• Has a deep commitment to facilitate research that is responsive to community priorities	2	–	100%	
• Has understanding of community organizations (e.g., capacity, culture, day-to-day life, needs)	1	96%		
• Has experience working with community organizations	1	86%		
• Has a big picture mentality	1	82%		
• Has experience disseminating programs with fidelity	1	82%		
• Has the ability to multitask	2	75%	82%	
**CRA roles and responsibilities**				
• Explaining to community and academic partners the steps involved in performing research in community settings	1	100%		
• Meets academic partners in person at some point during the study^a^	3	64%	77%	100%
• Exploring community interest and capacity to engage in research	1	96%		
• Assuring that communities’ input is considered in the study design	1	96%		
• Communicating pro-actively with partners to ensure project is on track	1	96%		
• Explaining to community and academic partners the importance of research	1	93%		
• Organizing the sharing of results with community partners	1	93%		
• Troubleshooting issues during project	1	93%		
• Facilitating planning meetings before grant is obtained.	1	93%		
• Explaining to all potential community and academic partners what the research network is (e.g., CAARN)	1	92%		
• Meets community partners in person at some point during the study^a^	3	71%	77%	90%
• Identifying partners (community and/or academic)	1	89%		
• Explaining to academic partners the context on which community organizations operate (e.g., Aging Network)	1	89%		
• Modeling communication strategies to request stakeholder input during planning meetings	1	86%		
• Assisting communications between academic and community members to assure that goals and expectations are accurately understood.	1	86%		
• Decisions regarding the CRA’s participation in community-academic team meetings be assessed on a case-by-case basis, depending on functionality of the relationship between the community and academic partners	2	–	84%	
• Providing assistance during grant application (Budget template, letters of support)	1	82%		
• Scheduling planning meetings before grant is obtained	1	82%		
• Scheduling research meetings between community and academic partners AFTER the grant is obtained^a^	2	71%	82%	
**Regarding the director of a research network like CAARN**				
• Has passion about the mission of the research organization	1	100%		
• Has good communication skills	1	100%		
• Is able to provide input regarding dissemination and implementation strategies^a^	3	71%	71%	100%
• Has good writing skills	1	96%		
• Has good problem-solving skills	2	n/a	95%	
• has knowledge of funding mechanisms	1	93%		
• Has experience in dissemination and implementation research	3	75%	76%	90%
• Is able to provide input on the scientific merits of the study, intervention design, and evaluation procedures^a^	3	71%	57%	90%
• Has experience in grant applications	1	89%		
• Identifies funding opportunities	1	89%		
• Provides assistance during development of grant application	1	89%	–	
• Has been successful obtaining NIH and other research funding	1	86%		
• Has good listening skills	2	n/a	86%	
• Participates in planning meetings	1	86%		
• Is experienced in community-based participatory research	1	82%		
• Has the ability to mediate conflict	2	n/a	81%	
**Regarding the purveyor(s) (i.e., the organization(s) that licenses and distributes the program to community agencies, once the program is proven.**				
• Organization’s goals align with goals of the research network	1	96%		
• Has capacity to disseminate new programs	1	96%		
• Is a trusted organization among community partners in the research network	1	96%		
• Distributes programs after they are proven	1	96%		
• Has experience disseminating programs	1	89%		
• Has experience delivering programs	1	89%		
• Provides input on existing needs	1	86%		
• Provides input on the packaging of evidence-base programs	1	86%		

Wording is equal to that used in the round when it achieved consensus

^a^Indicates rewording/clarification between Round 1 and Round 2

^b^Indicates rewording/clarification between Round 2 and Round 3

### Round 2

Round 2 of the survey was sent to the 28 individuals who completed Round 1; 23 (82%) completed Round 2. Table 1 shows the distribution according to type of panelist. Non-respondents include two individuals who were no longer working at their respective organizations and could not be reached for further rounds. Of the 48 items in Round 2, 11 achieved consensus and 37 did not. Of the 37, 12 did not meet the inclusion criteria for the subsequent round (> 50% of responders considered the item very/extremely important). Therefore, 25 items were included in Round 3 (Fig. 1).

### Round 3

Twenty of 23 individuals invited completed the survey sent for Round 3 (87%). Table 1 shows the distribution according to type of panelist within each round. Round 3 included 25 items, nine of which achieved consensus. Of the remaining 16, 11 achieved stability of ratings between Rounds 2 and 3 as determined by Spearman’s correlation test (Fig. 1). Authors MP and JM discussed the remaining five items along with associated comments; no new information was provided in Round 3 comments compared to Round 2; therefore, it was deemed that panelists would be unlikely to change opinion over subsequent rounds. Table 3 includes the elements that did not achieve consensus and the responses in each round, and the Supplemental Information file includes the comments received for each of these items.

**Table 3:** Items that did not achieved consensus

Non-essential elements	Percent of panelists that considered the element as essential			Spearman correlation (Round 2 vs Round 3)
	Round 1	Round 2	Round 3	
**Regarding the academic partners invited to participate in CAARN projects**				
• Principal Investigator (or their Mentor on the grant if they are junior) has experience doing dissemination and implementation research	50%	57%	55%	0.35
• Principal Investigator (or their Mentor on the grant, if they are junior) has experience doing community-based participatory research (CBPR)^a^	54%	48%		
**Regarding the community partners invited to participate in CAARN projects.**				
• Has experience with program evaluation^a^	32%	43%		
• Belongs to a larger network of agencies	41%	33%		
• Has previous experience participating in research activities^a^	25%	14%		
**Regarding the Community Research Associate (CRA)**				
• Has experience training people to serve as facilitators/leaders of health promotion programs	n/a	50%	40%	0.36
• Has research experience	54%	41%		
• Is assertive. (Assertiveness is defined as the capacity to effectively communicate both the community and academic partner’s needs. Also, speaking on behalf of someone)^b^	68%	64%	**75%**	0.22
• Has experience with community-based participatory research (community-based participatory research (CBPR) is defined as a collaborative approach to research that equitably involves all partners in the research process and recognizes the unique strengths that each brings)^a^	71%	55%	65%	0.09
• Has experience facilitating (or leading) evidence-based health promotion programs in community settings^a^	n/a	55%	55%	0.03
• Has experience establishing new partnerships or collaborations	n/a	71%	**75%**	0.22
**CRA roles and responsibilities**				
• Facilitates team meetings AFTER the grant is obtained (Facilitating refers to leading the meeting, or providing leadership without taking the reins)^a^	79%	64%	55%	0.66
• Provides input on materials that are created for the implementation or dissemination of the interventions^a^	68%	68%	**70%**	0.64
• Provides reminders to the academic and community partners about tasks and timelines related to the research process^a^	61%	64%	60%	0.52
• Confirms the delivery of payments, equipment, and other materials related to research activities to community organizations^a^	61%	36%		
• Provides input regarding the feasibility of the intervention and study design, implementation barriers and facilitators, and required elements for successful implementation^a^	64%	59%	**70%**	0.15
• Which of the following would be most beneficial to serve as home base for the CRA (Assuming that the home base provides all the necessary support (e.g., financial, supervision, management)^ab^	Academic 26%	Academic 27%	Academic 20%	0.45
	Community 12%	Community 18%	–	
		Either 31%	Either 75%	
	Other: 36%	Other: 22%	Other: 5%	
• CRA’s position includes non-research activities at the base organization in order to fully understand the community that they represent (meaning that they will be only part time with the research network)^a b^	0%	14%		
**Regarding the director of a research network like CAARN**				
• Has content knowledge that matches the focus of the research network^a^	68%	62%	65%	0.36
• Based at an academic organization in comparison to a community organization^a^	67%	57%	**75%**	0.3
• Has experience working in clinical settings (e.g., hospitals, clinics, community health center, or other healthcare provider organization)^a^	39%	24%		
**Regarding the dissemination organization(s)**				
• Has a commitment to disseminate new programs nationally (as opposed to only in the state)^a^	56%	48%	45%	0.34
• Has experience developing leader trainings for evidence-based programs^a^	78%	48%	55%	0.51
• Engages as a stakeholder in the research stages of the project before the program is proven^a^	75%	62%	65%	0.71
• Research network uses only one organization (as opposed to more than one) to disseminate the programs developed by the research network^a^	12%	14%		
• Has experience doing research	67%	33%		
• Be compensated for the time/effort spent in project activities performed in their role as a stakeholder	63%	38%		
• Addresses issues related to needs of researchers as creators of the product.		48%		

Wording is equal to the one used in the last round where the item was included

**Bold:** 70–79% of panelists indicated the element to be essential, but it did not achieved consensus

^a^Indicates rewording/clarification between Round 1 and Round 2

^b^Indicates rewording/clarification between Round 2 and Round 3

### Summary of all rounds

Consensus was achieved in 77 (67%) elements (Table 2). All of the elements regarding the overall network were considered essential: connects to a network of organizations; aligns projects to partners’ needs; designs for dissemination; involves partners who are interested in using the interventions in the future; establishes a relationship with a purveyor, and employs a research liaison (CRAs), an administrator, and an oversight committee.

About academic partners, 8 out of 11 elements were essential: should be committed to take projects to dissemination; consider community partners as equals on research team; have good communication skills; are open to feedback and learning new concepts; are successful in getting funding; and have some knowledge of designing for dissemination. Regarding community partners, 8 of 11 elements were consider essential: have supportive leadership and enthusiastic staff with capacity to participate in research; have strong ties to the community; are financially compensated by research project; and are invited to provide input in all phases of study.

The skills and characteristics that were considered essential for the CRA were as follows: good communication and organizational skills, positive attitude, ability to multitask, strong commitment to facilitating research that is responsive to community priorities, understands community organizations’ needs; has a big picture mentality; and has experience working with community organizations and disseminating programs with fidelity. Essential elements of the CRA’s role with community partners included exploring interest and capacity to engage in research; identifying potential community partners; assuring that community partners input is considered; modeling communication strategies, explaining steps in research; and organizing sharing of results with the community. Similarly, the CRA’s essential role with academic partners included explaining steps of community-based research, and explaining the contextual factors affecting community organizations. Essential responsibilities of the CRA in relation to the research partnership included explaining to community and academic partners the importance of research and the roles of CAARN in the research process; assisting with communication to make sure goals and expectations are clear between partners; communicating pro-actively; troubleshooting problems; providing assistance during grant writing; facilitating and scheduling planning meetings before a grant is obtained; and as needed, participating in team meetings after a grant is funded.

About the role of the network director, 16 of 21 elements were considered essential: has passion about mission; good communication, listening, writing, and problem-solving skills; is able to mediate conflict; has experience in getting research funding; has experience in community-based participatory research (CBPR) and dissemination and implementation (D&I) research; has knowledge of funding mechanisms and grant opportunities; and is able to provide input on study design and developing grant applications. About the purveyor, 9 of 17 elements were considered essential: it is trusted by community partners; its goals align with research network’s goals; it has capacity to disseminate new programs and has experience disseminating and delivering programs; it provides input to the research network on existing needs and on packaging of programs. Table 3 shows all items that did not achieve consensus, the directionality of responses in each round, and the Spearman correlation achieved between rounds. Table 4 shows those elements where the difference between the responses was statistically different according to the type of panelist.

**Table 4:** Items where responses differed by type of panelist

	Mean score [median] ***(range)*** ^**a**^			Mann-Whitney test (***p*** value)		
	Academic	Community	CAARN Staff / EC	Academic vs. community	Academic vs CAARN staff /EC	Community vs CAARN staff/EC
**Elements that achieved consensus in Round 1**						
• The research network has access to a network of community/local organizations	5.00 [5]	4.38 [4] *(Range 3–5)*	5.00 [5]	0.02*	1.00	0.08
• The CRA must have an understanding of community organizations (e.g., capacity, culture, day-to-day life, needs)	5.00 [5]	4.54 [5] *(Range 3–5)*	4.80 [5] *(Range 4–5)*	0.05*	0.33	0.56
• Director has been successful obtaining NIH and other research funding	4.70 [5] *(Range 3–5)*	4.00 [4] (*Range 3–5)*	4.80 [5] *(Range 4–5)*	0.01*	1.00	0.04*
• Community organization received financial support to compensate for time/staff that is dedicated to research related activities	4.60 [5] *(Range 3–5)*	4.08 [4] *(Range 3–5)*	4.20 [4] *(Range 3–5)*	0.03*	0.45	0.77
• Academic partner has good communication skills	4.80 [5] *(Range 3–5)*	4.38 [4] *(Range 4–5)*	4.40 [4] *(Range 4–5)*	0.02*	0.08	1.00
• Academic partner considers community partners as equals in the research team	4.80 [5] *(Range 3–5)*	4.31 [4] *(Range 3–5)*	4.40 [5] *(Range 3–5)*	0.03*	0.50	0.81
• Academic partner is open to receive feedback	4.80 [5] *(Range 3–5)*	4.31 [4] *(Range 3–5)*	4.80 [5] *(Range 4–5)*	0.03*	1.00	0.20
**Elements that achieved consensus in Round 2**						
• Principal Investigator (or their Mentor on the grant, if they are junior) has been successful in obtaining grant funding	4.22 [4] *(Range 3–5)*	4.43 [5] *(Range 3–5)*	5.00 [5]	0.55	0.03*	0.10
**Elements that did not achieve consensus after Round 3**						
• The CRA provides input regarding the feasibility of the intervention and study design, implementation barriers and facilitators, and required elements for successful implementation	3.56 [4] *(Range 2–5)*	3.338 [4] *(Range 3–5)*	4.60 [4] *(Range 4–5)*	0.7	0.09	0.01*
• Director should be based at an academic organization in comparison to a community organization	3.22 [4] *(Range 1–4)*	4.14 [4] *(Range 3–5)*	4.50 [4.5] *(Range 4–5)*	0.10	0.05*	0.65
• Dissemination organization has experience developing leader trainings for evidence-based program	3.22 [3] *(Range 2–5)*	3.86 [4] *(Range 3–5)*	5.00 [5]	0.09	0.01*	0.03*
• Principal Investigator (or their Mentor on the grant if they are junior) has experience doing dissemination and implementation research	3.22 [3] *(Range 2–4)*	4.43 [5] *(Range 3–5)*	3.50 [3.5] *(Range 3–4)*	0.01*	0.65	0.12
• CRA has experience establishing new partnerships or collaborations	3.67 [4] *(Range 2–5)*	4.71 [5] *(Range 3–5)*	4.75 [5] *(Range 4–5)*	0.03*	0.09	1.00

Abbreviations: *EC* Executive Committee

*Statistically significant

^a^Scale used for analysis of responses: 5 = extremely important, 4 = very important, 3 = moderately important, 2 = slightly important, 1 = not at all important

## Discussion

Since its inception in 2010, CAARN has participated in the development and testing of 32 interventions for older adults using the principles of “Design for dissemination [8],” resulting in three interventions that are now being disseminated across Wisconsin [2–4], and two that are being packaged for dissemination (that is, implementation manuals, pricing plans, train-the-trainer programs, and marketing materials are being finalized in collaboration with the purveyor) [1, 5, 6]. For these five interventions, the average timeframe from first research study to dissemination has been 6 years, which is significantly shorter than the average of 17 years that takes to translate 14% of original research into practice [17, 18].

The factors associated with the usual delay between development and adoption are diverse, including practitioners lack of awareness of the evidence, programs are not feasible for adoption and implementation in real-world settings or are not responsive to population needs, or programs cannot be sustained over time. To address some of these gaps, CAARN was established to accelerate not only the dissemination of new evidence, but also its adoption and implementation into community practice to improve the health of older adults. Funding from the University of Wisconsin – Madison and federal grants allowed CAARN to facilitate the development of programs before they secured grant funding, and build long-term relationships with stakeholders, which is considered as key to achieve the goal of hastening the uptake of research into practice.

The concept of community-academic partnerships is well described in community-based participatory research [19–21], as is the use of networks to facilitate translational research, for example, practice-based research networks (PBRN) that are formed by primary care clinicians [22, 23]. To our knowledge, CAARN is unique in the purposeful inclusion of translation for ultimate scale-up in its mission. In addition, CAARN purposefully includes a purveyor, whose roles and responsibilities maximize the possibility to disseminate new programs to partners whose goals, needs, and capacities support the adoption and implementation of the programs.

In this study, some of the elements that were considered essential to achieve CAARN’s goals were that the network has access to community organizations that represent potential adopters; alignment of goals among stakeholders; community partners having a history of strong, collaborative, and productive working relationships within their community; having personnel to support the network’s infrastructure (e.g., administrator, CRA); and the network having a commitment to move projects along the research continuum and make the resulting programs available to community partners. These elements are similar to those described as necessary in an infrastructure for stakeholder engagement by Nease et al. In their study, Nease et al. described the following domains: longstanding connections and relationships, having staff to support engagement activities, institutional culture to support engagement, the presence of relationships and engagement activities that are non-study dependent, and the use of systems promoting effective dissemination of study findings in conjunction with community partners [24]. A difference between the work of Nease et al. and ours is that CAARN’s primary goal is to facilitate not only the development but also the ultimate scaling-up of evidence in a way that reduces the time to dissemination and broad adoption of the evidence-based program. As such, CAARN’s infrastructure is based on the capacity to engage academic partners, community partners, and purveyors to maximize the ability to design for dissemination [8].

The use of liaisons (such as the CRAs) is well described in the literature for community-based participatory research, although their specific roles and responsibilities vary by study [25, 26]. Liaisons tend to work to support engagement activities within single studies rather than multiple studies simultaneously [24, 27]. Gaglioti et al describe the liaisons as network facilitators for PBRNs, where the liaisons come from within the network and their role is to understand the values and preferences of the organization, and support their decisions to participate in new projects; this is done either by serving as an advisor, or as cross-pollinator of ideas [28]. In our study, the liaison (CRA) supports building relationships between new partners, which includes incorporating new members to the network. The CRA’s role is to facilitate the communications between community and academic stakeholders in order to build and maintain their relationships as research partners. Our study provides detailed characterization of the specific skills necessary to serve as a CRA, the specific activities that the CRA performs as teams cohere, and how the role varies according to the research stage and other contextual factors.

As mentioned before, the CRA has been a community representative, who has been hired by the purveyor to split their time between research activities with CAARN, and delivery and implementation of evidence-based programs with the purveyor [1]. Of note, only 14% of panelists thought it was essential that the CRA be hired by and part of a community organization. The panelists were asked what type of institution should serve as the home base for the CRA, with home base defined as the institution that provides all the necessary support (e.g., financial, supervision, and management). The Delphi results showed that the CRA could be based at either a community or an academic organization. Comments, and the results of other items, clarified that regardless of who hires the CRA, it would be essential that the CRA understand the community and be visible to community organizations, and that for certain projects, having a physical location within the community might be an important aspect to facilitate the creation of new partnerships.

Other Delphi items considered essential included the CRA should have the communities’ benefits as a priority; the CRA should have the expertise to communicate effectively with all the stakeholders; and the CRA should facilitate all research meetings between community and academic partners prior to the grant submission. However, it was not essential that the CRA facilitate community-academic research team meetings after a grant is obtained if the academic and community partners have developed functional, mutually beneficial relationships. The panelists suggested that after a grant is funded, the CRA’s specific activities would depend on the type of project; the preferences of the academic and community individuals; the length, stability, and functionality of the community-academic partnership; and the anticipated needs of the projects. Regardless of frequency of contact, the CRA continues to troubleshoot, ensure community input is recognized, and facilitate planning for future grants to move an intervention along the translational spectrum towards dissemination and implementation in practice.

Several of the items in the Delphi related to previous expertise of the CAARN staff. The panelists’ indicated that the only essential elements in terms of previous expertise for CRAs were “having experience working with community organizations,” and “having experience disseminating programs with fidelity.” The comments suggested that overall knowledge in certain aspects (e.g., research) would be helpful but was not a pre-requisite, since it can be the subject of trainings based on specific project needs. Similar responses were seen for items related to previous experience of the purveyor and the community and academic partners. For the purveyor, it was deemed essential that the organization has experience disseminating and delivering programs, but not essential that it has experience doing research. Nor was it essential for the community partners to have previous experience doing research. For the academic partners, while knowledge of D&I principles was considered essential, it was not essential that they have actual experience with either D&I or CBPR research. Comments revealed the importance of the facilitative function of the CRAs or other members of the CAARN team, including the director, in overcoming lack of experience on the part of the community or academic partners, or the purveyor.

The need for expertise was highlighted for the role of director who, according to our panelists, needed to have experience in dissemination and implementation science, community-based participatory research, and obtaining funding from the National Institutes of Health (NIH) and other funding agencies. Overall, the panelists agreed that the director’s role was to maximize the chances of garnering funding for projects by providing input into research design and grant applications and helping to identify funding sources. The director’s role in facilitating translation was also deemed essential. Although other studies do not clearly delineate the role of the director, they acknowledge that scientific expertise and leadership committed to the organization’s goals are necessary elements to promote collaboration and subsequent dissemination of results [24]. Our findings add to this by demonstrating a clear role for expertise in helping design fundable studies and providing input regarding dissemination and implementation strategies to promote translation into practice. The expertise and experience of the director can compensate for an academic partners’ lack of D&I or CBPR expertise. Ultimately, having an experienced and knowledgeable director enables investigators without that methodologic experience to take advantage of CAARN to move their research closer to real-world application.

The dissemination organization, also called purveyor, refers to the organization whose role is to disseminate the new behavior change program with fidelity and provide support for organizations to adopt and implement the program [24, 29–31]. In this study, panelists affirmed that having strong partnerships with organizations interested in disseminating final products is essential to successful translation of interventions developed through research networks; this is in line with results from other studies [24, 32]. Panelists agreed that more than one organization could serve as a purveyor. Panelists indicated that the ideal purveyor should have a good reputation among community end-users, have experience in delivering and disseminating programs, provide input on the best way to package programs for dissemination, and have the capacity to scale up programs. These characteristics are in line with the implementation strategies employed by successful purveyors [31]. New to the literature, our study highlights that the majority of the panelists (65%) agreed that the relationship with the purveyor can be established before the intervention is proven. However, some comments indicated hesitation in setting up an early relationship with a purveyor, because the best fit in terms of purveyor organization might not be known in early stages; instead, it might best be determined as the research process proceed over time.

The strengths of this study include that panelists were a diverse group of stakeholders who have been involved with CAARN studies in the past as academic or community partners, CRAs, or members of CAARN’s EC. These experiences provided panelists with an understanding of the elements that are essential to achieve the goals of a research network like CAARN, therefore providing an array of perspectives for this study.

### Limitations

Our results are most applicable to research networks working with community partners who are organized around a specific topic (e.g., healthy aging); therefore, our findings may be less generalizable to practice-based research networks or to other networks that have different goals and stakeholders. The limitations of our study include those inherent to Delphi studies: (1) there is substantial variability in the methodology used to determine consensus and the parameters used to decide the retention of items between rounds [15] and (2) the attrition rate between rounds, which affects the final composition of the panel of experts. The first Delphi survey included a number of items and required a substantial time commitment on the part of panelists. Considering the complexity of the network, and the number of stakeholders involved, the large number of items used was necessary to obtain a comprehensive view of all the personnel and their roles and responsibilities. To minimize attrition, we provided several reminders in between rounds and provided an incentive for completion of Round 3. Additionally, the research team, with input from the EC, developed a comprehensive list of items for consideration, but there may have been additional items that were essential but were not included. Another limitation is that not all panelists had participated in projects that had reached the dissemination phase, which might have affected the final rating of items related to dissemination and skewed the results to items important in early stages of research. Lastly, the choice of a cut-off of 80% to determine consensus is arbitrary. In other studies, the cut-off has ranged from 70 to 80% [16], with a higher cut-off being favored for more homogeneous groups of panelists [33]. There were five items for which 70 to 79% of panelists deemed the item to be extremely or very important to CAARN’s success (Table 3), such as CRA having experience in establishing new partnerships, CRA providing input on the feasibility of the intervention and the packaging of materials for dissemination, and the Director being based at an academic organization. Note should be taken of these elements where a substantial majority considered the items to be highly important to CAARN’s success, and they were not essential based on our arbitrary cut-off point.

## Conclusion

This study provides a set of guiding concepts deemed essential to the success of a community-academic research network that can intentionally serve as a pipeline to dissemination. The essential elements meet the priorities of adopters, implementers, and end-users and provide the necessary support to community and academic partners to develop and test interventions with those priorities in mind. Finally, this study delineates essential elements related to engaging purveyors in a community-academic research network. One of the key findings of our study is that our panelists agree that diverse personnel in the network’s infrastructure are essential to facilitate the relationships between academic partners and community organizations and that each stakeholder (e.g., community partner, academic partner, purveyor, community research associates, and director) has specific roles and responsibilities to facilitate the research processes and packaging and hand-off for dissemination. This information is currently used to guide hiring and training of new CAARN staff and can be used by other organizations/entities to build their research network toward hastening the development, testing, dissemination, and ultimate scale-up of feasible, effective interventions to improve health.

## Supplementary Information

**Additional file 1: Supplemental Information.** This table contains the comments received for those items that were considered not essential according to the results of Table 3.

## Data Availability

The datasets generated and analyzed during the current study are not publicly available due to privacy reasons but are available from the corresponding author on reasonable request.

## Abbreviations

CAARN: Community-Academic Aging Research Network
EC: Executive Committee
CRA: Community Research Associate
US: United States of America

## References

[1] Mahoney JE, Pinzon MM, Myers S, Renken J, Eggert E, Palmer W (2020). The community-academic aging research network: a pipeline for dissemination. J Am Geriatr Soc..

[2] Gretebeck KA, Blaum CS, Moore T, Brown R, Galecki A, Strasburg D, Chen S, Alexander NB (2019). Functional exercise improves mobility performance in older adults with type 2 diabetes: a randomized controlled trial. J Phys Act Health.

[3] Chewning B, Hallisy KM, Mahoney JE, Wilson D, Sangasubana N, Gangnon R (2020). Disseminating tai chi in the community: promoting home practice and improving balance. Gerontologist. Gerontologist..

[4] Brown HW, Braun EJ, Wise ME, Myers S, Li Z, Sampene E, Jansen SM, Moberg DP, Mahoney JE, Rogers RG (2019). Small-group, community-member intervention for urinary and bowel incontinence: a randomized controlled trial. Obstet Gynecol.

[5] Mora Pinzon M, Myers S, Jacobs EA, Ohly S, Bonet-Vázquez M, Villa M, Castro A, Mahoney J (2019). “Pisando Fuerte”: an evidence-based falls prevention program for Hispanic/Latinos older adults: results of an implementation trial. BMC Geriatr.

[6] Koltyn KF, Crombie KM, Brellenthin AG, Leitzelar B, Ellingson LD, Renken J, Mahoney JE (2019). Intervening to reduce sedentary behavior in older adults - pilot results. Health Promot Perspect.

[7] Crombie KM, Leitzelar BN, Almassi NE, Mahoney JE, Koltyn KF (2019). Translating a “Stand Up and Move More” intervention by state aging units to older adults in underserved communities: Protocol for a randomized controlled trial. Medicine (Baltimore).

[8] Brownson RC, Jacobs JA, Tabak RG, Hoehner CM, Stamatakis KA (2013). Designing for dissemination among public health researchers: findings from a national survey in the United States. Am J Public Health.

[9] Boulkedid R, Abdoul H, Loustau M, Sibony O, Alberti C (2011). Using and reporting the Delphi method for selecting healthcare quality indicators: a systematic review. PLoS One.

[10] Murphy MK, Black NA, Lamping DL, McKee CM, Sanderson CF, Askham J, Marteau T (1998). Consensus development methods, and their use in clinical guideline development. Health Technol Assess.

[11] Custer RL, Scarcella JA, Stewart BR (1999). The Modified Delphi Technique--A Rotational Modification. J Vocational Tech Educ.

[12] Hasson F, Keeney S, McKenna H (2000). Research guidelines for the Delphi survey technique. J Adv Nurs.

[13] Keeney S, Hasson F, McKenna H (2006). Consulting the oracle: ten lessons from using the Delphi technique in nursing research. J Adv Nurs.

[14] Slade SC, Dionne CE, Underwood M, Buchbinder R (2014). Standardised method for reporting exercise programmes: protocol for a modified Delphi study. BMJ Open.

[15] von der Gracht HA (2012). Consensus measurement in Delphi studies: Review and implications for future quality assurance. Technological Forecasting and Social Change..

[16] Diamond IR, Grant RC, Feldman BM, Pencharz PB, Ling SC, Moore AM, Wales PW (2014). Defining consensus: a systematic review recommends methodologic criteria for reporting of Delphi studies. J Clin Epidemiol.

[17] Balas EA (1998). From appropriate care to evidence-based medicine. Pediatr Ann.

[18] Green LW (2014). Closing the chasm between research and practice: evidence of and for change. Health Promot J Austr.

[19] Israel BA, Parker EA, Rowe Z, Salvatore A, Minkler M, Lopez J, Butz A, Mosley A, Coates L, Lambert G (2005). Community-based participatory research: lessons learned from the Centers for Children's Environmental Health and Disease Prevention Research. Environ Health Perspect.

[20] Wallerstein N, Duran B (2010). Community-based participatory research contributions to intervention research: the intersection of science and practice to improve health equity. Am J Public Health.

[21] Israel B, Schulz A, Parker EA, Becker AB, Allen AJ, Guzman JR, Minkler M, Wallerstein N (2008). Critical issues in developing and following CBPR principles. Community-Based Participatory Research for Health : From Process to Outcomes.

[22] Green LA, Dovey SM (2001). Practice based primary care research networks. They work and are ready for full development and support. BMJ.

[23] Heintzman J, Gold R, Krist A, Crosson J, Likumahuwa S, DeVoe JE (2014). Practice-based research networks (PBRNs) are promising laboratories for conducting dissemination and implementation research. J Am Board Fam Med.

[24] Nease DE, Burton D, Cutrona SL, Edmundson L, Krist AH, Laws MB, Tamez M (2018). “Our lab is the community”: Defining essential supporting infrastructure in engagement research. J Clin Transl Sci.

[25] Hoeft TJ, Burke W, Hopkins SE, Charles W, Trinidad SB, James RD, Boyer BB (2014). Building partnerships in community-based participatory research: budgetary and other cost considerations. Health Promot Pract.

[26] Skizim M, Harris N, Leonardi C, Scribner R (2017). Academic-community partnership development to enhance program outcomes in underserved communities: a case study. Ethn Dis.

[27] Medina NG, Baez LS, Mendez LB (2018). Collaborative transdisciplinary research in a small institution: challenges and opportunities. Inform Sci.

[28] Gaglioti AH, Werner JJ, Rust G, Fagnan LJ, Neale AV (2016). Practice-based research networks (PBRNs) bridging the gaps between communities, funders, and policymakers. J Am Board Fam Med.

[29] Franks RP, Bory CT (2015). Who supports the successful implementation and sustainability of evidence-based practices? Defining and understanding the roles of intermediary and purveyor organizations. New Dir Child Adolesc Dev.

[30] Graff CA, Springer P, Bitar GW, Gee R, Arredondo R (2010). A purveyor team's experience: lessons learned from implementing a behavioral health care program in primary care settings. Fam Syst Health.

[31] Proctor E, Hooley C, Morse A, McCrary S, Kim H, Kohl PL (2019). Intermediary/purveyor organizations for evidence-based interventions in the US child mental health: characteristics and implementation strategies. Implement Sci.

[32] Fixsen D, Naoom S, Blase K, Friedman R, Wallace F (2005). Implementation research: a synthesis of the literature.

[33] Croes KD, Jones NR, DuBenske LL, Schrager SB, Mahoney JE, Little TA, Burnside ES (2020). Core elements of shared decision-making for women considering breast cancer screening: results of a modified Delphi survey. J Gen Intern Med.

