# Supplementary material for: Essential elements to “design for dissemination” within a research network—a modified Delphi study of the Community-Academic Aging Research Network (CAARN)
Source: Implement Sci Commun. 2021 Feb 12;2:18. doi: 10.1186/s43058-021-00122-z (PMC7881665; doi:10.1186/s43058-021-00122-z)
Supplement: Supplementary file 1 — Additional file 1: Supplemental Information. This table contains the comments received for those items that were considered not essential according to the results of Table 3. [file 43058_2021_122_MOESM1_ESM.pdf]

Supplemental Information: Comments received for items considered non-essential.

Contents

Academic Partners 2
Community Partner 3
Community Research Associate (CRA) 5
The Director 16
Dissemination organization(s) or purveyor 18

Non-essential item as written in the last round.	Comments
Academic Partners	
• Principal Investigator (or their Mentor on the grant if they are junior) has experience doing dissemination and implementation research	• Beneficial, but probably ok if they didn't.<br>• The CRA can help facilitate this<br>• Can be taught/ other types of research are important<br>• Someone should have that perspective to coach the process along.<br>• If they don't get the word out about the success of their project, they will not receive peer recognition and approval plus there is the possibility of no future funding.<br>• The principal investigator is responsible and should be well versed in doing dissemination and implementation research.<br>• Important for all staff to understand what the dissemination and implementation entails
• Principal Investigator (or their Mentor on the grant, if they are junior) has experience doing community-based participatory research (CBPR)	• Would be great if they did, but I'd also like to take junior researchers and have CAARN excite and teach them about it, so that they get jazzed and eager. Everybody has to start somewhere.<br>• If the researcher has a project that they feel strongly in, they will work with the proper authorities to make sure the project will be effective.<br>• Communication, planning, and development is critical...not so much the prior experience<br>• Training might be sufficient at first<br>• This can be taught<br>• Without this, it is unlikely the project will be successful due to the unique characteristics of partnership needed for success.<br>• Without some experience with community-based participatory research, the principal investigator could make mistakes which would cause problems for the research.<br>• Vital to overall success of program

Non-essential item as written in the last round.	Comments
**Community Partner**	
• Has previous experience with program evaluation	• Makes it easier but that is something that can be taught<br>• That can be taught.<br>• Beneficial, not necessary.<br>• This could be part of the training.<br>• It would be helpful, but it would not be a reason to exclude potential partners.<br>• If the CRAs do their job, this is not as crucial.<br>• That skill and process can be taught - since every data collection process is different it would have to be learned on a case by case basis<br>• Ongoing evaluation is necessary, even if periodic, for new partnership development and funding justification.<br>• At the end of the day this needs to be done to keep it going.<br>• Important for several types of proposals
• Belongs to a larger network of agencies	• Depends on the research<br>• Depending on project needs<br>• Not a deal breaker<br>• Great tool to learn from others and get the word out about your project/research<br>• Depends on funding<br>• It can help get the research out to a greater audience, but is not necessary.<br>• Helpful for grantsmanship<br>• Adds to potential dissemination<br>• Could be beneficial, but not necessarily important. Reaching the smaller organizations with less support would also be important.<br>• Want a broad network of potential program providers, broad network of potential participants, connections with payers, connections with health systems, connections with funders.<br>• Dissemination can only go far with a network to disseminate in.<br>• As a senior center director who has not been trained in the geriatric field, I have found networking to be extremely helpful and a tremendous source of information.

• Has previous experience participating in research activities	• That can be taught and role of CRA to help facilitate that<br>• Training could be built into the research project.<br>• Helpful, but can leave that to the research network arm.<br>• If you make it too important, critical people will not be eligible.<br>• Isn't that what the training is for? To bring up to speed those that don't?<br>• Everyone has to start somewhere - with CAARN support I would hope that communities without experience could get their sea legs<br>• Certainly beneficial, not necessary.<br>• As long as there is the willingness on the community partner to offer a program that is being researched, they will do what is required upon receiving the proper procedures from the CRA & researcher.<br>• they would learn<br>• Important to Understand Research

Non-essential item as written in the last round.	Comments
**Community Research Associate (CRA)**	
• CRA has experience training people to serve as facilitators/leaders of health promotion programs	• This is assuming one model of health promotion programs. You can learn and understand the components and challenges of leader training without having done the training yourself.<br>• Much more or much less depending on the nature of the current project.<br>• Again, it helps that the CRA has an understanding on how past programs were packaged and disseminated to best practices used in leader trainings<br>• Training and communication are not the same thing.<br>• This can be taught<br>• Would be great to have but seems less critical than the others experiences listed<br>• If the CRA is supposed to be facilitating communication with academic and community partners, I do not see where experience in training facilitator/leaders is important.<br>• It is important for a person with those skills to be on the team but the CRA does not need to be that person.<br>• Having that grassroots experience will assist in knowing what is realistic for leaders/participants<br>• This is important, because of the dissemination and implementation component of program development. Developing leader training curriculums is a crucial component of facilitating a project through all the necessary stages. Even the pilot stage needs training components.<br>• Working with volunteers and training volunteers, yes. This is what keeps the programs running.<br>• This would be an attractive option for academics<br>• If this experience is lacking, the overall success of leaders will be impacted.<br>• The CRA has to be able to train community partners and their facilitators in proper instructional presentation to maintain program fidelity required for evidence-based status.
• Has research experience	• Need to understand research and believe in it. Would be nice to have some research experience but not too much as this might shift the commitment from community responsiveness to research. I think this might undermine their effectiveness<br>• It's definitely important, but I feel like that's something that can be taught more easily than the interpersonal skills, and project management and organizing skills. Am open to the possibility that I am 100% wrong on that.

	• It depends on the role of the CRA. If the role of the CRA is to build bridges, extensive research experience is not important. If the role of the CRA is to enroll participants in research, research experience is very important.<br>• Because the academic side is representing the research component, I'm not sure it's necessary, but certainly helpful for the CRA to have experience in research. If they did not have experience, I believe they could still be effective by learning about research as they went.<br>• This can be obtained on the job. It's more about the soft skills- empathy, communication, lived experience etc.<br>• Some research knowledge is important, but that can be learned on the jog, relationship building is more important and just understanding how both sides work.<br>• This depends on the complexity of the CRA's role in the project.<br>• I think working with others and having experience in communication is more important.<br>• Again, research experience is helpful when explaining some of the nuances to community partners, but I feel that the CRA could learn as they participate as well...so I don't think research experience is as critical as the ability to communicate and the CRA's interest in learning something new.<br>• Trying to organize/advocate for research projects to be started requires some background<br>• I think leadership experience is more important than research experience.<br>• It would help to know basic research concepts.<br>• A basic understanding of research is needed in order to accurately and effectively coach community partners through the process. Expanded knowledge can be obtained with project experience, but a basic level must be there to start.<br>• This position likely would involve working remotely or similar, making it challenging to gain much on the job training<br>• The CRA needs to be aware of the requirements of research studies either as a researcher or community partner.
• Is assertive. (Assertiveness is defined as the capacity to effectively communicate both	• I think one of your questions is problematic because of word choice. According to the dictionary, assertive means "having or showing a confident and forceful personality" Do I think the CRA should be confident or forceful? NOT AT ALL IMPORTANT - in fact, forceful is undesirable for someone who is meant to build bridges. *(this response was edited for brevity)*<br>• In my opinion assertive is a negative word. If a CRA is necessary I would think it would be important for a CRA to be an effective leader.

the community and academic partner’s needs. Also, speaking on behalf of someone)	• Assertiveness is less important being a good listener and communicator.<br>• The CRA needs to be able to stand up and represent either the academic side to the community partners, or the community partners to the academic side, if the situation ever occurs.<br>• Assertiveness is important to get things done, but that term itself is problematic. It's an art, knowing when to be assertive and when to step back.<br>• Unclear how assertiveness is a quality that would assist with this work.<br>• Gently assertive but not pushy.<br>• This is a tough job and is sometimes difficult to help people on both sides see the world from the other point of view. At least requires great persistence.<br>• Note that I don't consider "assertive" to be a negative term. CRA has to be able to assertively but sensitively deal with disrespectful researchers or community partners, and to get people to honor their time commitments, etc., amongst other tasks.<br>• The CRA needs to be assertive enough to effectively communicate both the researcher and community partner's needs.<br>• Assertive is an essential skill but there are strategies for keeping folks in line with the goals<br>• Assertiveness is needed in representing community and academic needs in projects. The CRA has an advocacy role where this is needed.<br>• May just be a terminology thing. I'm interpreting 'assertive' as 'tenacious'<br>• In collaborative situations, it is important to acknowledge what is communicated from others before clearly communicating what the CRA understands to be important.<br>• The CRA needs to constantly remind others to keep her/him up to date.<br>• Addressing issues between the community partner and academic partner requires assertiveness to communicate and resolve any concerns that may arise during the projects.<br>• It is important for the CRA to take in all the information and make educated decisions which may require being assertive in what direction the research will take.<br>• Researchers, due to funding requirements, are usually on a strict timeline which need to be adhere to. Thus the CRA needs to be aware of this and work with a community partner to see that programs can be accomplished with in the required time frame. Also needs to impart to the community partner about the need to uphold to the requirements of the study.<br>• As a liaison, you would need to be observant of situations and be able to communicate them if even conflict or upsetting situations and or positive and important changes needed etc.

• Has experience with community-based participatory research (Community-based participatory research (CBPR) is defined as a collaborative approach to research that equitably involves all partners in the research process and recognizes the unique strengths that each brings.)	• In this type of research, the role of the CRA will be defined by the investigator or a steering committee.<br>• Would be nice to have some research experience but not too much as this might shift the commitment from community responsiveness to research. I think this might actually undermine their effectiveness<br>• I think it would be very important for them to UNDERSTAND what CBPR is, but not necessarily that they have experience in this area. I think they should have experience working with community partners so they are able to represent them well.<br>• Considering how CAARN has worked - the person should have at least been a participant so they can speak from personal experiences (challenges and successes) and anticipate obstacles.<br>• Important to the overall success of programs<br>• I think the CRA should specifically get training in CBPR but does not need to come with that experience necessarily<br>• It's important to be able to walk the community partners through the process and be able to explain effectively how it works.<br>• Understanding how to work with the community in research can be of benefit to the position.<br>• The CRA's understanding of the partners' roles is imperative in providing communication and delineating expectations.<br>• Research with communities/people would be essential. Because the work with this cause is in the community, for the community<br>• a basic knowledge should exist in order to accurately coach partners through the process.
• Has experience facilitating (or leading) evidence-based health promotion programs in community settings	• I don't think you need to have lead a health promotion program to understand the dynamics involved, the needs of communities and the requirements of research. More important to have the skills to bring the parties together in a positive direction.<br>• Would be real helpful, but I don't think it's a deal breaker. Can go and observe, read about it, learn the all-important "you're a facilitator, not a Teacher" philosophy.<br>• Much more or much less depending on the nature of the current project.<br>• If they have the other necessary characteristics - leader training could be built upon but some experience would be helpful<br>• It helps when it comes to packaging up the programs for dissemination, if the CRA understands how past programs have been taught and organized

	• Assertiveness rather than leadership is more important.<br>• "It is important, but not necessary. The community partner will ideally have that experience, and the CRA's role should be to facilitate the partner's communication of that experience to the team<br>• It is important for a person with those skills to be on the team but the CRA does not need to be that person.<br>• Knowing or working with groups of communities folks at any level, but especially in a facilitator/ leader role would be a bonus.<br>• I think having the experience of working with community members and being on the community partner side of things is very important for the CRA to be able to properly represent the community partner side of things.<br>• That was a helpful clarification - I believe I was moderate last time regarding "leader" in general.<br>• I vacillated between very and extremely important. I think this job requires many skills and the ability to cross worlds, translate, understand multiple perspectives.<br>• If the CRA has done this before, they can provide real world implementation solutions that bridges the academic-community divide.<br>• leadership experience is harder to come by and seems less easy to teach<br>• Many of these projects need to be formulated into an evidence-based program and knowing how these programs work in a community setting is important for smooth implementation.
• Has experience establishing new partnerships or collaborations	• Helpful skill but not absolutely necessary - can train someone on engaging new programs. Networking is a skill all its own<br>• Much more or much less depending on the nature of the current project.<br>• The CRA should concentrate on communication.<br>• Can be built upon if they have some experience<br>• It depends on CAARN's focus and how robust current partnerships are- not sure of the demand for new relationships<br>• Need to be able to hear from ALL communities to truly understand the needs, which means continuously establishing new partnerships and collaborations<br>• Since the CRA will be establishing new relationships with partners throughout their entire job, it is very important.<br>• Definitely needed for grow. Can't keep going back to aging network, so has to be someone who is a true collaborator, someone who knows how to cross cultures of organizations, professions, communities.

	• Making connections and facilitating community-based academic research projects is only possible with the ability to establish and maintain partnerships and collaborations. This is the most important component, in my opinion.<br>• This is a challenge and requires some knowledge of how different communities interact and organize<br>• This is more key to the position than some of the above factors.<br>• seems like that is the primary job of the CRA - to establish and nurture new partnerships - not sure that experience as opposed to training is the most important thing<br>• Many partnerships and collaboration would be necessary in this role<br>• I think this sort of experience could be very valuable. It is the sort of skills that are central to effectiveness<br>• Relationships and good communication is key to capitalizing on a community's strength and collaborating partners.<br>• CRA needs to be aware of available community partners either by networking, and working with agencies which services older adults such as ADRD's, senior centers and other
• CRA facilitates team meetings AFTER the grant is obtained (Facilitating refers as leading the meeting, or providing leadership without taking the reins)	• Depends on the skill set of the groups that are coming together. One or the other may have someone skilled in facilitation that they would prefer in this role<br>• This should be responsive to each situation rather than a generalization about the CRA.<br>• CRA should help early on but as project progresses CRA likely does not need to play as big of a role.<br>• It's not clear that who facilitates the meeting matters so much so long as people trust that person<br>• Especially if there has been revisions in the original program required to secure funding.<br>• Helps to establish a timeline for the project<br>• Same rationale as earlier statement<br>• The CRA could gain help with the next research project.<br>• The CRA can be extremely helpful in setting the stage and getting team meetings set on the most effective track. The CRA has the experience in how this works well, from best practices in other successful projects.<br>• the CRA should play a lead role in the meeting as this is likely the position the CRA would be in during the conduct of the project<br>• This is so dependent on the specific situation.

	• The CRA is the connector...and they need to be present at meetings.<br>• It helps establish the CRA's role of the communicator between the two entities.<br>• Depends on experience of partners and stage of research.
• Provides input on materials that are created for the implementation or dissemination of the interventions	• It is a team effort by the research team, community partners and CRA. All inputs are needed.<br>• again - seems like you are asking whether a CRA can serve every role of a community collaborator, which doesn't seem like the purpose of the CRA. The CRA should help academicians get feedback from their community collaborators; the CRA might not have the same perspective as they do<br>• would be nice to have the community provide input<br>• More important for the community partner to provide input on materials.<br>• They have experience in this and knows from bumps previous ones ran into what training might help to avoid those occurring in this one.<br>• This was a helpful clarification. They don't necessarily need to be content experts but should know how to design the materials to be effective for the trainer, manager etc.<br>• Part of the cras responsibility is to assist with D&I to move it to a purveyor. Their experience with packaging materials will be valuable, especially to ensure continuity between programs.<br>• Assuming the CRA has insight into what is important to the community, their concerns, practices and understands the research.<br>• The CRA would know over time what is most effective for dissemination from experience on multiple studies<br>• This assumes that the CRA knows insights about the audiences who will receive the materials.<br>• The CRA's role should be to make sure the materials are easy to understand.<br>• Important, with the perspective of what has worked with other programs.<br>• The CRA understands the community needs/implementation process in rolling out programs. Their input on materials is very important for the success of the program.<br>• It's important for the CRA to share their knowledge of what has worked and what has not in past projects to help researchers decide best methods.
• Provides reminders to the academic and community partners about	• Seems like this responsibility should fall on the research team<br>• this could be handled by another staff member<br>• it depends on the project management capacity of the academic partner- sometimes cutting out the middleman can be more efficient<br>• someone needs to keep the project on the schedule, on task to be completed on time.

tasks and timelines related to the research process	• the academic and community partners should be able to accomplish this without CRA<br>• seems this job could be done by anyone<br>• Comments extremely/very<br>• this should be a part of ongoing efforts to assure essential communication.<br>• this depends on the project and the PI, but the CRA should be making sure it is happening one way or the other.<br>• the CRA again keeps community partners on task as the community partners' role is much greater than participating in a particular research project. The timeline reminders improves overall project success.<br>• both entities are very busy with other jobs, i think this is one of the key roles of the CRA<br>• always helpful in every project.<br>• it seems as though this is needed to ensure everyone stays on track.<br>• this is vital to keep things going. People all have busy lives and this sort of support helps everyone, prevents problems<br>• keeping to the timeline is very important to<br>• important that the entire team is on the same page and reminders of tasks and timelines will be vital to the overall success of the program<br>• that should be one of the CRA's top priorities.
• Confirms the delivery of payments, equipment, and other materials related to research activities to community organizations	• Could be handled by another staff member, but CRA should be knowledgeable about these events<br>• Community partners may also have systems in place...<br>• This depends on the project, PI. They could assure that it is happening, but as long as they are checking in with the team members, they will know if and when there is an issue where they need to step in.<br>• With or without the CRA, these deliveries should be confirmed.<br>• Could this be delegated?<br>• Could be anyone<br>• I think this fall under the role of whoever is overseeing the grant- so not necessarily the CRA.<br>• Nothing is more distressing to community than to feel like you have to fight for what was agreed on. This can also be quite complex.<br>• This is part of key communication components.

	• Resources are critical, and if they are not delivered appropriately, then this can ruin relationships. So, having some guarantee of this is critical for the network overall<br>• Confirms is much better than assures - because it just providing oversight and follow through rather than being part of the process which can't always happen per regs<br>• Important that CRA can advocate for communities if they don't feel comfortable advocating for themselves.<br>• Again, in the agreement, following through on payments/and expectations is just expected and assures a good working relationship.
• Apart from the CRA’s role facilitating communications regarding study design between partners, how important is it to the effectiveness of a research network like CAARN that the CRA provides their input regarding the feasibility of the intervention and study design, implementation barriers and facilitators, and required elements for successful implementation	• The CRA's position is to help implement by providing communication between the different groups in the research program. Planning should only be to aid in the communication process.<br>• Depending on who all is on the team, other members may have more knowledge in that area relates to the research but then we need to make sure it will work in "real life" for our parners and participants.<br>• It is a team effort by the research team, community partners and CRA. All inputs are needed.<br>• To the degree that the CRA is persuasive, the CRA's ideas should be taken into account.<br>• Someone with that expertise should be on the team but does not have to be the CRA<br>• CRA is going to have to sell it to both partners, so she needs to be involved in the designing so it's never ever a "I don't know why they designed it that way" situation. Presumably she will also have experience with previous research projects so that she will be able to best advise on what she knows will be challenging elements and what will clear the path more easily.<br>• It is critical they share their experience/expertise working on multiple projects to inform best practices.<br>• They will have the best sense of this over time across studies, and should provide that information to both the community and academic partners<br>• The knowledge and experiences of the CRA should be able to provide valuable input in this area.<br>• It's important because the CRA will have more experience as to what has worked well or not well with other projects. However, its most important that the CRA facilitate the community partners input, because each community is individual and the partners know their community best.<br>• Important that this role not just be viewed as a facilitator but also a contributor

	• I don't know that all study design should be influenced by CRA/community, some elements of study design require hard stops or a decision not to use that design based on scientific validity<br>• CRA can contribute insight but the job is really to bring others perspectives to the table<br>• This CRA input will minimize obvious stumbling blocks at a high level and improve implementation and progress.<br>• It is important for the CRA to share their experience of what has worked well and did not work well in similar projects/communities in the past.<br>• CRA will have a good overall picture and it will be important to communicate that vision to all of the partners and that will help to have successful implementation<br>• Important to ask questions of academic and community partners, share experiences of similar projects and point out identified challenges.
• Which of the following would be most beneficial to serve as home base for the CRA (Assuming that the home base provides all the necessary support (e.g. financial, supervision, management, etc).	• ***Comments in favor of Academic***<br>o This is where the projects are begun and run from.<br>o The bigger dollars will likely come from grants and partnerships - therefore the University should provide the overhead<br>• ***Comments in favor of Community***<br>o because research is less common at community level, may be more critical to have a research minded individual on sight<br>o They need to be a part of the community network in order to understand the needs of the community and engage community partners.<br>o This would help the imbalance between academic and community organizations.<br>o In the balance between needs of researcher and research institution and needs of community, when conflict occurs, I believe CAARN's philosophy has come down firmly on community side. Hence, I'd prefer they stay connected to the community side, out closer to the groups that will test the intervention, implement the intervention and the end-users.<br>• ***Comments for Either***<br>o Depends entirely on the project. But they need to be present and visible in both places<br>o It depends on the context- if there are strong relationships and no competition between the community orgs, than that is ideal because then it looks less like self-interest of academics. However, if there is competition, then the academic becomes the neutral space.<br>o it's not clear to me that this matters<br>o I don't think the location matters, as long as the CRA has adequate resources to do their job.

	○ It depends on the primary role. If community already trusts research, then having CRA at academic location would make supporting academics easier. If community doesn't trust research, CRA should be located in the community.<br>○ The agency whether academic or community based would need to support the CRA
• CRA's position includes non-research activities at the base organization in order to fully understand the community that they represent (meaning that they will be only part time with the research network).	• As long as the CRA comes in with the knowledge, skills, and experiences necessary to complete this job, unnecessary for them to be doing something else simultaneously.<br>• Likely to have time and focus diluted<br>• There are many ways to stay connected<br>• I think it is part of their job to go learn how each community operates (part of the relationship building, partnerships, etc)- but that is encompassed into the CRA role. I think CRA can be a Full-Time position.<br>• Unless you have a CRA for each community, i don't see how this question makes sense. Is the CRA supposed to have a presence with every community agency?<br>• The CRA sounds like a full-time job already - being part of all the other components. Perhaps 90% CRA duties with 10% other?<br>• Good to not be "renting space" at the community org, but to be integrated with its day-to-day work so that they can see the end of the rainbow for their project and what it takes to implement and maintain support for programs.<br>• I think the CRA position should be solely focused on the research project. If they work on non-research activities it may be difficult to recruit for position as well as keep that persons expectations clear<br>• If they are housed in a community based organization, again, they need to be aware of the organization's priorities or focus, but if the CRA job involves working with community partners and the academic partners, this detailed information isn't as important<br>• In order to understand the needs of the community, the CRA must be an active part of the community network, as needs are always changing.<br>• This answer depends on the nature of the research. A CRA may become stronger in their role if they engage in non-research activity.<br>• This seems like a good idea but not essential<br>• In order to understand the needs of the community, the CRA must be an active part of the community network, as needs are always changing.<br>• The CRA has to understand both worlds

Non-essential item as written in the last round.	Comments
**The Director**	
• Has content knowledge that matches the focus of the research network	• I think people are pretty flexible in their ability to adapt to different age people<br>• I value their expertise - but also more from a research angle rather than clinical angle<br>• I think that is something that can be learned and built upon based on need<br>• Nice, but not critical. If they've worked in Research, CBPR and D&I that works for me.<br>• Important for project prioritization purposes.<br>• Again, content knowledge is important to oversee/take responsibility and accountability for projects--i.e. Prioritizing resources, funding, etc.<br>• This should receive strong consideration.<br>• speaks to alignment of mission<br>• A general content knowledge is important to provide guidance
• Based at an academic organization in comparison to a community organization	• There are several homes for a director, doesn't need to be academic<br>• it could work either way<br>• as long as they have resources and contacts in the academic organization i don't think it has to be based in an academic organization<br>• pros: looks good for grants, cons: maybe not as trusted by community<br>• prestige, gravitas, resources, cache, connections.<br>• they need to have the connections to academic expertise and resources.<br>• assuming that an academic organization means they have more experience than someone working in a community organization, that would be important. More important than what type of organization they should be based in, would be the experience they had. And how funding was handled at the different organizations.<br>• the ability to obtain funds may be greater in an academic setting...resources may be accessible to a higher level.<br>• The director would be more effective working from the academic organization.
• Has experience working in clinical settings (e.g.	• As long as there are others on staff with that experience, it is not as necessary for the director to have.

hospitals, clinics, community health center, or other healthcare provider organization)	• Helpful, but the academic partner will have this knowledge, as well as a member or two on the executive committee if needed.<br>• not sure why clinical setting matters, health doesn't have to be based in a clinic<br>• It depends on the specific project.<br>• This should be most influenced by the mix of research projects. Director's experience should be like the mix.<br>• Not sure why this would be important considering it does not represent neither the community partner side nor the academic side.<br>• it depends on the focus of the research network<br>• Meh. We've all been patients too. We all know that no clinical setting has time or expertise to facilitate their patients' behavior change.<br>• It's important but also depends on the types of interventions. I think their role needs to speak to some avenue of health care and clinical work - but that experience may be 10 years ago!<br>• It's good to have a wide perspective not just a clinical view<br>• Since these are health-based interventions, the director needs to know enough about the problems first-hand to help guide the partnering

Non-essential item as written in the last round.	Comments
Dissemination organization(s) or purveyor	
• Has a commitment to disseminate new programs nationally (as opposed to only in the state)	• Some may be great interventions that are needed and valuable, but a market that requires licensing just won't support, so can't ALWAYS promise national dissemination if it would be at odds with business model.<br>• Depends on the funding source<br>• If you're talking about only one dissemination organization than commitment to broader reach is necessary.<br>• Not all interventions need to have national reach to be effective<br>• It's helpful to demonstrate larger potential impact in a grant<br>• Depends if you have research ready to go to the national level or not<br>• I think state only is ok, with the acknowledgement that the program could find different dissemination organizations in each state for the same program. For some programs- this may be preferred.<br>• Definitely a benefit but many programs never make it that far<br>• Local entities are going to be responsible for providing programs to communities, the organization needs to keep abreast and translate national information to local organizations<br>• For federal grants to be successful, ideally reach is beyond a single state<br>• What works in Wisconsin should work nation-wide or even world-wide. Sharing what we learn is important to creating a better world.<br>• This would help when seeking national funding. This would do more good.<br>• With all the resources given to create a new intervention, it would be most efficient to share those resources nationally instead of each state having to create on their own.
• Has experience developing leader trainings for evidence-based programs	• They could outsource that<br>• Training the trainer would be important.<br>• This may be helpful.<br>• Depends on whether the intervention requires a leader training<br>• That is a big part of the dissemination process.<br>• Crucial function of a purveyor.

	• It depends on what the program is. It is important that they have the capacity to train whoever is wanting to replicate the program.<br>• Important to be effective in dissemination and leader training is an important aspect of that<br>• Since they will be the ones doing this.<br>• As the "parent organization" it would be important to understand and implement appropriate trainings
• Engage as a stakeholder in the research stages of the project before the program is proven	• Depends on whether it is known at the outset what organization will be in the best position to disseminate.<br>• Depends on what is being disseminated.<br>• Many grants will ask for this<br>• To disseminate and purvey statewide or nationwide dissemination - resources for staff time are needed - as it goes above and beyond what they are already doing.<br>• If you want the organization to be committed, they have to also feel the commitment.<br>• The organization needs to be aware and be included as a consultant<br>• They would provide valuable input as a community representative, and as a representative of the dissemination organization to determine what they're looking for.<br>• Demonstrates long-range thinking on the proposal, and clear trajectory
• Research network uses only one organization (as opposed to more than one) to disseminate the programs developed by the research network.	• No idea on this one. Maybe it's nice to have options? Don't want the purveyor org to be cocky, or controlling, or hold a project hostage.<br>• this sounds very inflexible<br>• different interventions require different dissemination plans!<br>• Get it out in a way that assures effective adoption.<br>• The more dissemination the better if it can be managed well<br>• Although there could be benefit to this, I do think different projects need different purveyors and that would be ok.<br>• Once again, it depends on the research.<br>• This would be the most effective and efficient.<br>• Helps to simplify process<br>• Consistency and regular communication through one organization is best and improves overall success
• Has experience doing research	• It would be important for them to understand the research put into the project, but if their role is solely dissemination, having experience doing research is not a necessity.

	• They should have an understanding, but they should focus on the D&I more than needing research experience.<br>• Or willing to learn. Have to appreciate the design, need for fidelity, evidence-based, key elements if going to effectively disseminate and hold program provider partners to the fidelity.<br>• More important to understand research and the requirements of dissemination.<br>• This only matters if you are doing dissemination research<br>• Dissemination partners rely on the information that the research team provides<br>• Once the programs have been deemed evidence-based, the need for the parent organization to do research is not as important<br>• Training would be important to help these organizations with the research.<br>• Some situations could change my mind on this topic.<br>• Important enough that they understand the importance of research- but considering they're not doing any research and just get the project after the research has been completed, not that important.<br>• Helpful for continuing good relationship between all partners
• Be compensated for the time/effort spent in project activities performed in their role as a stakeholder	• While compensation is nice, most organizations should not do the research for that reason.<br>• It depends on the services provided/time involved, expertise required<br>• Depends on how the funding for that organization is structured- if dissemination is the entire mission and there is need for compensation then it is important<br>• Depends on whether they stand to gain financially from their participation overall<br>• All players should be adequately compensated for their time.<br>• Time and perspective are valuable to the entire process.
• Addresses issues related to needs of researchers as creators of the product.	• May be helpful<br>• Always important to be flexible and look outside the box<br>• Need to be open-minded and therefore creative.<br>• This adds to the commitment and "buy-in" of the organization.
